# The Complex Relations Between Wisdom and Significant Life Learning

**DOI:** 10.1007/s10804-017-9261-1

**Published:** 2017-04-09

**Authors:** Shih-ying Yang

**Affiliations:** National Chi Nan University, 1 University Rd., Puli, Nantou, 54561 Taiwan

**Keywords:** Wisdom, Learning, Life experience, Chi square, Aiken’s *H*, Questionnaire, Taiwanese Chinese

## Abstract

This study explores the various relations between two important processes in life-span development: wisdom and learning from significant life experience. A diverse sample of 375 individuals from Taiwan, culled from an initial sample of 475, completed a sequence of three questionnaires that asked them to describe their most significant life learning, the wisdom they had displayed, and the relation between the two. The 375 participants specified one of five relations: (a) their most significant life learning led to their display of wisdom (*n* = 191, 51%); (b) the two were unrelated (*n* = 91, 24%); (c) their display of wisdom led to their most significant life learning (*n* = 67, 18%); (d) the wisdom displayed was part of their most significant life learning (*n* = 20, 5%); (e) their most significant life learning was part of the wisdom they displayed (*n* = 6, 2%). These results suggest that wisdom and significant life learning are seen as related in various ways. The findings shed light on how wisdom and the learning acquired from significant life experiences foster individual development.

## Introduction

Wisdom and learning from significant life experience pertain to positive human development (Baltes 1987; Jarvis 2009). This study explores various relations between these two important processes in life-span development.

### Wisdom and Learning from Life

Although scholars have yet to reach consensus on the definition of wisdom, many wisdom theorists have proposed that wisdom and the learning acquired from important and meaningful life experiences are related (Ardelt 2010; Bluck and Glück 2004; Booker and Dunsmore 2015; Erikson 1982; Kramer 2000; Smith et al. 1994; Sternberg 2007; Yang 2014a).

Empirical studies exploring the relations between wisdom and life learning have been lacking. However, results from a few empirical studies do point to certain connections between wisdom and lessons learned from life experiences. Exploring the wisdom coming from life’s challenges, Choi and Landeros (2011) interviewed 18 low- and moderate-income older adults who were nominated by their service providers as being wise. The lessons that these participants drew from challenging life experiences were congruent with their views about the ultimate qualities of wisdom and their actions and behaviors. Bluck and Glück (2004) interviewed 86 Caucasian Germans regarding their experience of wisdom, as defined by the participants’ subjective definition of wisdom, and found that the majority of their participants (*n* = 67, 77.9%) had learned meaningful lessons from the same experience. To explore whether learning acquired from important life experiences can foster wisdom, I (Yang 2014b) conducted a series of interviews and surveys that spanned more than 2 years. Working with eight Taiwanese leaders in higher education, I recorded the leaders’ accounts of the lessons they learned during a 6-month leadership experience and the components of wisdom that they displayed in applying those lessons to other areas of life in the following one-and-a-half years. I then tested these findings by administering an inventory consisting of the lessons learned and the components of wisdom to another 94 leaders in Taiwanese higher education twice within 8 months. The results showed that participants’ responses to both the lessons learned and the components of wisdom increased with leadership experience and that these increases were strongly and positively correlated, suggesting that learning from important and meaningful life experiences can foster wisdom. Altogether, these findings suggest that complex and various relations may exist between wisdom and significant life learning.

### Wisdom

The study takes into account (a) that seeing wisdom as a process has been useful in exploring related topics in the past (Yang 2014b), and (b) Choi and Landeros’ (2011) criticism that “a serious deficit in previous research on wisdom is that it focused more on the thinking and feeling aspects than the doing aspect of wisdom” (p. 595), and uses a process view of wisdom to explore possible complex relations between wisdom and learning from significant life experience. In this view, wisdom is defined as a process which emerges in a specific context within a specific period of time through the interaction between an individual and a real-life situation that he or she faces. The process has three structural components: cognitive integration, embodying action, and positive effects for oneself and others. Although wisdom may take a different and unique form in different contexts, the real-life process we call wisdom begins when a person cognitively integrates elements that are ordinarily considered separate or incompatible to form a benevolent idea or a vision. The process continues when the person takes actions to embody his or her integrated idea or vision, and is completed when the embodying actions generate positive effects for the acting person and for others (Yang 2008a, 2014a).

#### Cognitive Integration

Wisdom develops from extraordinary judgment or unusual effort; it seldom arises from merely following existing rules and norms. A person may need to integrate different perspectives and divergent or even conflicting ideas to solve problems, transcend suffering, resolve conflicts, heal wounds, and bring peace. Even though the actual content to be integrated will differ in different circumstances, wisdom is characterized by a benevolent idea that comes from integrating different, even opposing, ideas. The integration of life and death in Solomon’s judgement of the two prostitutes fighting over a child (*1 Kings 3:16–28*), the integration of sensual indulgence and the severe asceticism in Buddha’s Middle Way (Salman 2012), the integration of public affairs and personal ethics in Confucius’ endeavor to bring harmony to warring states (*Confucius: The Analects, Book II: 1, 3*), and the integration of the glory of God and the suffering of the poor in Mother Teresa’s charity (Yang 2013) are a few examples of ideas or visions that arise from such cognitive integration.

#### Embodying Action

Having an integrated humane idea is just the beginning. As we may observe in real life, wisdom also involves deeds that embody the integrated idea or vision. Thus, embodiment—actions taken to embody the integrated idea in real-life situations—is an essential part of wisdom. As Choi and Landeros (2011) asserted, “The pragmatic qualities of wisdom dictate that it must be practiced in the real world” (p. 595). The actual actions differ from case to case; broadly, however, wisdom demands that we do the right things to implement the integrated idea, and that we do those things in the right ways. This is consistent with the arguments of other scholars, such as Birren and Fisher (1990) who observed that *“*being wise and displaying wisdom reflects forms of behavior that are admired, condoned, and encouraged” (p. 318). Ardelt (2010) proposed that “wisdom is inherently concerned with ethical and moral conduct and the pursuit of social justice for all” (pp. 312–313), and Bierly et al. (2000) argued that “[w]isdom is not merely a result of inquiring and reflecting on the relationship between self and society, but it is also the embodiment of action taken to transform self and society towards a better whole” (p. 603).

#### Positive Effects for Oneself and Others

A necessary test of whether the integrated idea is embodied in the right ways is if that embodiment generates positive effects for both oneself and others. Hence, the other essential feature of wisdom is its positive influences. An individual’s embodiment of the integrated idea should generate a positive influence for the self by, for example, resolving real-life problems, gaining emotional or psychological rewards from accomplishing a self-defined life mission, living a better life, or earning the respect and admiration of others (Yang 2008b, 2013).

The actions that individuals take to embody their ideas also often affect their surrounding others; persons whose embodiment generates positive effects not only for themselves but also for others at the individual, familial, communal, societal, or even global levels are often credited with greater wisdom (Yang 2008b, 2011, 2013, 2016). Despite the best intentions to help others, a person whose actions benefit only him- or herself is seldom credited with great wisdom. As Birren and Fisher (1990) argued, what is most wise is what is good for the greatest number of people in the long run.

Empirical results support this view of wisdom. Terrini (1994) showed that vignettes containing consequences of an individual’s decision were rated significantly higher for wisdom than those without consequences. Bluck and Glück (2004) found that, in most of their “experienced wisdom” narratives, the outcomes were more positive than the eliciting events, and many involved helping and supporting others. In Choi and Landeros’ study (2011), many of the participants’ descriptions of their wisdom involved actions that benefited others. Moreover, Yang (2008a) found that when participants were asked to nominate individuals they considered wise, they described cognitive integration, actions, and positive effects to justify their nominations. Further, when wisdom nominees were asked to describe their own wise decisions and actions, their narratives contained these three components (Yang 2008b, 2011). In addition, independent evaluators gave higher wisdom scores to narratives that were seen as embodying all three components (Yang 2008b).

### The Wisdom of Ordinary People

Both the renowned and the ordinary can display wisdom. Randall and Kenyon (2004) argued that “wisdom is not something reserved for extraordinary souls” (p. 339). Bluck and Glück’s (2004) study exploring ordinary people’s experience of wisdom showed that participants of all ages were able to describe their experience of wisdom in 2 min, and the majority of the descriptions were of reasonable length and dealt with significant events in life. Confucius, Buddha, and Mother Teresa were not initially renowned for their wisdom when they began to form their cognitive integrations. They were credited with wisdom after their endeavors generated positive effects for many others. Hence, I argue that ordinary people can display wisdom, although many episodes of their wisdom may remain unknown to us. Using the process model of wisdom, everyone who strives to live a meaningful and satisfying life can display wisdom if he or she integrates separate or incompatible ideas cognitively, embodies the integrated idea through actions, and generates positive effects for the self and others.

### Significant Life Learning

Most of us experience daily life in a continuous flow of perceptions, and hence are rarely aware either of the specific experience we have from mundane encounters with the world or of the knowledge and skills we learn from this process. The experiencing process often remains at a preconscious level as long as what we experience can be assimilated easily into our cognitive structures or meaning system (Jarvis 2009). What we learn through such daily participation in a human community may be hard to articulate (Polanyi 1958). We may become aware of some of the knowledge, skills, life philosophy, and behaviors we have tacitly acquired through life experiences when we try to, for example, orient friends who have returned to our home country after living abroad for a prolonged period of time.

An *experience* is defined as “encountering, undergoing, or living through things in general as they take place in the course of time” (Gove 1986). One is more likely to be conscious of a life experience if it does not meet one’s expectations (Jarvis 2009). If the experiencing individual, after reflecting upon the raw experience, is able to derive lessons from the specific experience, then the experience becomes a learning experience (Kolb 1984). Many studies have shown that learning from life experiences is the primary and most valued form of learning in adulthood (Dewey 1938/1997; Jarvis 2009; Merriam and Clark 1993).

Carl Rogers (1983) noticed that a learning experience becomes significant when what is learned is related to personal development. In this case, not only does the lesson learned affect the learner personally, but the learner values it subjectively. It may include a new way of learning, the expansion of knowledge and skills, or major changes in behaviors, attitudes, beliefs, and perspectives (Merriam and Clark 1993). In the study reported here, significant life learning is defined as the process, and as the outcome of the process, whereby new ways of learning, expanded knowledge and skills, or changed behaviors, attitudes, beliefs, and perspectives are derived through the transformation of significant life experience (Jarvis 2009; Kolb 1984).

### Five Relations Between Wisdom and Significant Life Learning

The study proposed that at least five possible relations exist between wisdom and significant life learning. First, *significant life learning fosters wisdom*—the abilities required to display wisdom can be developed by significant life learning. A previous study (Yang 2014b) showed that learning acquired from important and meaningful life experiences can develop the abilities that foster manifestations of wisdom, particularly the ability to integrate perspectives, take actions to embody ideas and visions, and examine and evaluate effects one’s actions have for oneself and others. Second, *wisdom fosters significant life learning*. Wise decisions, actions, or life management can direct our lives onto a more positive path, bring different experiences to us, and lead us to encounter different sets of events, thus opening new doors for learning. Hence, the reverse of the first relation—that wisdom can foster significant life learning—may be true as well. Third, *significant life learning is part of wisdom*. Sometimes the process of wisdom can take a long period of time (Yang 2013, 2016), and a person may acquire important and significant life learning within the wisdom process. Thus, significant life learning may occur as part of the wisdom process. Fourth, *wisdom is part of significant life learning*. It is also possible that some learning processes can be lengthy, even lifelong, and a person may display wisdom within that lengthy process. Thus, wisdom may be part of significant life learning. Finally, it is also possible that there is *no relation* between wisdom displayed and significant life learning.

### The Present Study

In this study, I used a series of three questionnaires to explore the possibility of various relations between wisdom and significant life learning. I hypothesized that the five relations would be evident in participants’ recollection of their significant life learning and their displays of wisdom.

### Methods

I asked participants to describe what they learned from their most significant learning experience and display of wisdom to ascertain the relation between wisdom and significant life learning.

#### Materials

The study involved three questionnaires that were written in Chinese and consist of open-ended and close-ended questions. The questionnaires were based on the process definition of wisdom (Yang 2008a), and also followed Park and Helgeson’s (2006) suggestions that reports of growth made in the context of cognitive processing might be more authentic and “some combination of open-ended and close-ended questions could be helpful in capturing actual growth” (p. 794).

All three questionnaires asked the participants to provide basic information about themselves. In addition, the first questionnaire, the *Survey of Learning from Life Experiences*, consists of eight open-ended questions and one 7-point Likert-scale question. The open-ended questions asked participants to describe their most significant learning experience in life, what they had learned from this life experience, the insight/new idea they gained from this learning experience, the actions they took to carry out what they learned, and the positive effects that their actions generated for themselves and others. The scaled question asked participants to what extent they agreed that what they learned brought them closer to their conceptions of wisdom (1 = “strongly disagree,” 7 = “strongly agree”).

The second questionnaire, the *Survey of Display of Wisdom*, consists of nine open-ended questions and one 7-point Likert-scale question. The open-ended questions asked participants about the wisest thing they had done in their lives, the event that led to such a display of wisdom, the idea that had motivated their wise action, the actions they took to carry out their idea, and the positive effects that their actions generated for themselves and others. The scaled question asked participants to what extent they felt that their display of wisdom was truly wise (1 = “strongly disagree,” 7 = “strongly agree”).

The third questionnaire, the *Survey of the Relation between Significant Life Learning and Wisdom*, consists of three open-ended questions and one six-item selection question. The first two questions asked participants to briefly repeat their most significant learning experiences and the wisest thing they had done, respectively. The third question asked participants to specify the relation between the two with one of the following statements: (a) the display of wisdom led to the most significant life learning, (b) the display of wisdom was part of the most significant life learning, (c) no relation between the two, (d) the most significant life learning led to the display of wisdom, (e) the most significant life learning was part of the display of wisdom, and (f) other. The last question asked the participants to provide an explanation for the relation they specified.

The order of the first two questionnaires was determined based on the results of a pilot study conducted to test the survey questions. After both the first two questionnaires were piloted in different sequence, it seemed that asking about wisdom first often resulted in many participants responding with the same experience for both questionnaires, indicating that their two experiences were the same. I thus put the *Survey of Learning from Life Experiences* first to avoid this stronger priming effect of the second questionnaire. In addition, the detailed descriptions that I requested through these very specific questions served to verify the participants’ most significant life learning and displays of wisdom, and to deter them from using invented stories to complete all three questionnaires.

#### Procedures

I posted an advertisement written in Chinese on the PTT Bulletin Board System, a non-commercial and general country-wide website in Taiwan, which has more than 1.5 million registered users and is the largest terminal-based bulletin board system in Taiwan. The advertisement was posted for 1.5 years and asked for participants who were 19 years and older and would like to share their life experiences and lessons learned from life by completing three questionnaires. After participants responded to the advertisement, I sent the three questionnaires through email in sequence: The second questionnaire was given after the participants had returned the first questionnaire, and the third questionnaire was given after the participants had returned the second. The reason for the strict sequence of the questionnaires was to avoid participants selecting both experiences to fit a certain relation prior to responding to the questionnaires. Participants received a monetary reward of $300 NT dollars (roughly $10 U.S. dollars) for completing all three questionnaires.

I also sent questionnaires to colleagues residing in the southern, central, and northern parts of Taiwan who gave out the three questionnaires in the same sequence with the same monetary reward. Most questionnaires were collected through email, and a few of them were collected through postal mail.

#### Participants

Four hundred and seventy-five citizens of Taiwan completed all three questionnaires in Chinese. Among them, 316 (67%) were females. The participants’ age ranged from 19 to 92 (*n*_*19*_ = 3, 0.6%; *n*_*20*–*29*_ = 275, 58%; *n*_*30*–*39*_ = 71, 15%; *n*_*40*–*49*_ = 36, 8%; *n*_*50*–*59*_ = 68, 14%; *n*_*60*–*69*_ = 12, 3%; *n*_*70*–*79*_ = 8, 2%; *n*_*91*–*92*_ = 2, 0.4%), with an average age of 32.9. Most of them had a college-level education (*n*_*below elementary*-*level education*_ = 1, 0.2%; *n*_*elementary*-*level education*_ 14, 3%; *n*_*junior*-*high*–*school-level education*_ = 16, 3%; *n*_*senior*-*high*-*school-level education*_ = 41, 9%; *n*_*college*-*level education*_ = 292, 61%; *n*_*master*-*level education*_ = 103, 22%; *n*_*doctor*-*level education*_ = 8, 2%). Two hundred and sixty-nine participants (57%) indicated that they did not believe in any religion, while the remaining 206 (43%) participants believed in either folk religion (*n* = 69, 15%), Christianity (*n* = 55, 12%; *n*_*Protestant*_ = 49, 10%; *n*_*Catholic*_ = 6, 1%), Buddhism (*n* = 40, 8%), Taoism (*n* = 39, 8%), or other (*n* = 3, 0.6%). Participants came from 18 provinces, constituting most of the territory of Taiwan, with only two island provinces left without any participant in this study.

To determine if my initial sample was representative of the population of Taiwan, I consulted the *2014 Statistic Yearbook* (Ministry of the Interior of Taiwan 2015). Results of the Chi square comparisons between the demographic distribution of the initial sample of 475 and the estimated percentages of educational level (among the 25–29 years old), gender, and age group in the total population showed that the sample of 475 differed from the total population of Taiwan on gender (*X* ^2^_(1)_ = 51.27, *p* < .001), age (*X* ^2^_(5)_ = 656.93, *p* < .001), and education (*X* ^2^_(1)_ = 209.96, *p* < .001). However, results of *post hoc* tests (Sharpe 2015) showed the two did not differ significantly on the percentage of those aged 30–39 (Std. Residual = −1.09, *p* = .28), 50–59 (Std. Residual = −0.39, *p* = .70), and 70 and above (Std. Residual = −2.84, *p* = .02), and on those with a junior high level (Std. Residual = 0.23, *p* = .82) and college level of education (Std. Residual = −0.10, *p* = .92). Thus, the recruited sample of 475 had significantly more women, more participants aged 19–29 years old (Std. Residual = 24.49, *p* < .001), more with an elementary-level education and below (Std. Residual = 11.37, *p* < .001), and with a graduate-level education (Std. Residual = 6.50, *p* < .001), but significantly fewer participants with a senior high school level of education (Std. Residual = −6.19, *p* < .001), fewer participants who were 40–49 years old (Std. Residual = −4.59, *p* < .001), and 60–69 years old (Std. Residual = −5.15, *p* < .001). Percentages of religious believers were not compared with the total population for lack of exact statistics: The *2014 Yearbook* registered 4% of the total population as believers of a certain religion. However, the *2006 Yearbook* stated that 35% of Taiwan’s population considered themselves Buddhist, 33% were Taoist, and many people also considered themselves both Buddhist and Taoist (cited in American Institute of Taiwan 2011).

### Data Analysis

After the researchers ascertained that the questionnaires did not contain any confidential information, the 475 questionnaires were sent to three groups of analysts/raters to ascertain the validity of responses. Each group consisted of two or three analysts/raters. The analysts/raters had at least a 10-hour training for analyzing and rating interview transcripts of wisdom nominees (Yang 2008b) before they proceeded to analyze these data.

#### Relation Between Wisdom and Significant Life Learning

The first group of analysts/raters read the participants’ responses on all three questionnaires in sequence. They were first asked to check for inconsistent answers to repeated questions, particularly whether the respondents’ most significant learning experience and their display of wisdom described in the third questionnaire were consistent with what they had described in the first and second questionnaires. They were then asked to give *a relation rating* based on how logical and reasonable the relation between the most significant life learning and the display of wisdom was as indicated and explained by the participants (1 = “strongly disagree that it’s reasonable and logical,” 7 = “strongly agree”).

#### Analysis and Rating of Components of Wisdom

The second group of analysts/raters analyzed and rated the participants’ responses on the second questionnaire, the *Survey of Display of Wisdom*, based on the process definition of wisdom. To ascertain the validity of ratings, the analysts/raters were asked to first analyze then rate the responses.

The analysts/raters were first asked to analyze the participants’ responses on a thematic/content basis for the different categories (Creswell 1998). These categories included (1) participants’ idea that had motivated their wise action (i.e., “What was the specific idea the participant described at the moment s/he made the wise decision or took the wise action?”), (2) their cognitive integration (i.e., “What were the different things or separate elements the participant linked/integrated in his/her idea?”) (3) their embodying actions (i.e., “What were the actions taken by the participant to implement his/her idea?”), (4) the positive effects the embodying actions generate for oneself (i.e., “What was/were the positive effect(s) that the participant’s action generated for him- or herself?”), and (5) the positive effects the embodying actions generate for others (i.e., “What was/were the positive effect(s) the participant’s action generated for others?”). Working independently, the analysts/raters were asked to extract relevant statements from each participant’s response.

The analysts/raters were then asked to give *wisdom component ratings* (1 = “strongly disagree,” 7 = “strongly agree”) showing the extent of their agreement that the participants (1) had a motivating idea (i.e., “The participant described a specific idea that motivated him/her to make the wise decision or take the wise action.”), (2) showed cognitive integration in their ideas (i.e., “The idea described by the participant reveals a linkage/integration of different/separate things/entities/ideas.”), (3) demonstrated embodying actions (i.e., “The participant took actions to implement his/her idea.”), (4) generated positive effects for him- or herself through actions (i.e., “The participant’s actions generated positive effects for him- or herself.”), and (5) generated positive effects for others through actions (i.e., “The participant’s action generated positive effects for others.”).

#### Categorizing and Rating of Life Learning

To explore the domains of significant life learning, the third group of analysts/raters categorized and rated the participants’ responses on the first questionnaire, the *Survey of Learning from Life Experiences*. The analysts/raters were asked first to categorize participants’ significant life learning into one of the following ten categories, based on whether the main theme of the life learning was related to (a) *self*, (b) *everyday life management*, (c) *life philosophy*, (d) *overcoming external difficulty and challenge*, (e) *formal education and extra-curriculum activities*, (f) *career decisions and life direction*, (g) *work*, (h) *romantic relationship*, (i) *family, and* (j) *friendship and other interpersonal relationship*. In case some participants’ experiences could fall into more than one category, the analysts/raters were asked to select the one category that best captured the most important theme of those participants’ responses. The analysts/raters then rated the responses based on their own judgment whether the participants’ life learning involved wisdom (1 = “did not involve wisdom at all,” 7 = “definitely involved wisdom”).

#### Construct Validity and Inter-rater Reliability

The three questionnaires have construct validity since they asked questions concerning components of wisdom based on the process theory of wisdom (Nunnally and Bernstein 1994). Inter-rater reliability was calculated for all rating groups. Aiken’s *H* (Aiken 1985), a reliability coefficient that evaluates internal consistency of ratings on ordinal scales and ranges between 0 (no internal consistency) and 1 (perfect internal consistency), was used to calculate inter-rater reliability.

## Results

Results include participants’ self-rating, analysts/raters’ wisdom component and relation ratings, inter-rater reliabilities, the relations between wisdom and significant life learning as specified by participants, and the main themes of significant life learning.

### Ratings

#### Analysts/raters’ Ratings

Table 1 lists the means, standard deviations, and Aiken’s *H* coefficients from analysts/raters’ ratings for the 475 participants’ responses concerning the motivating ideas, cognitive integration, embodying actions, the positive effects for oneself, the positive effects for others, the relation between the displayed wisdom and significant life learning, and whether participants’ life learning involved wisdom based on analysts/raters’ own judgment.

**Table 1:** Ratings for Components of Wisdom and Relation between Wisdom and Significant Life Learning

Rating categories	*M*	SD	Aiken’s *H*
Motivating ideas	6.74	0.62	0.93*
Cognitive integration	6.16	1.31	0.94*
Embodying actions	6.88	0.42	0.96*
Positive effects for oneself	6.86	0.44	0.95*
Positive effects for others	5.98	1.60	0.91*
Relation	6.17	1.98	0.98*
Whether life learning involved wisdom (analysts/raters’ own judgment)	6.38	0.85	0.89*

*p < .05

These results show that the inter-rater reliabilities were quite high. In general, the analysts/raters agreed that most participants’ displays of wisdom involved the core components of wisdom, that the relation between wisdom and learning chosen by the 475 participants was reasonable and consistent with the descriptions and explanations that the participants provided, and participants’ life learning involved wisdom based on analysts/raters’ own judgment.

#### Participants’ Self-ratings

The mean of the 475 participants’ self-ratings for whether what they learned made them closer to their conceptions of wisdom was 5.80 (SD = 0.96); the mean rating for whether their display of wisdom was wise was 6.03 (SD = 0.98). This suggests that, in general, participants agreed that their significant life learning brought them closer to their conceptions of wisdom and that their displays of wisdom were, in fact, wise.

### Selecting the Analytic Sample

There were three criteria for selection into the analytic sample: (a) the participants’ responses were consistent on repeated questions; (b) the responses on the second questionnaire were deemed wise by the analysts/raters based on the process definition of wisdom (i.e., the responses had a mean wisdom component rating across analysts/raters equal to or above 4 on every wisdom component); and (c) the responses indicated a logical and reasonable relation between the displayed wisdom and the significant life learning (i.e., had a mean relation rating across analysts/raters equal to or above 4 on the specified relation between wisdom and significant life learning).

Three-hundred and seventy-five participants were selected as the analytic sample. Among them, 250 (67%) were females. Most of the participants a had college-level education (*n*_*below elementary*-*level education*_ = 1, 0.3%; *n*_*elementary*-*level education*_ = 13, 3%; *n*_*junior*-*high*-*school level of education*_ = 14, 4%; *n*_*senior*-*high*-*school level of education*_ = 35, 9%; *n*_*college level of education*_ = 228, 61%; *n*_*master level of graduate education*_ = 78, 21%; *n*_*doctor level of graduate education*_ = 6, 2%). Their ages ranged from 19 to 92, with an average age of 33.23 (*n*_*19*_ = 3, 0.8%; *n*_*20*–*29*_ = 215, 57%; *n*_*30*–*39*_ = 54, 14%; *n*_*40*–*49*_ = 25, 7%; *n*_*50*–*59*_ = 58, 15%; *n*_*60*–*69*_ = 11, 3%; *n*_*70*–*79*_ = 7, 2%; *n*_*91*–*92*_ = 2, 0.5%). Two hundred and ten participants (56%) indicated that they did not believe in any religion, while the remaining 165 (44%) participants believed in folk religion (*n* = 53, 14%), Christianity (*n* = 42, 11%; *n*_*protestant*_ = 38, 10%; *n*_*Catholic*_ = 4, 1%), Buddhism (*n* = 35, 9%), Taoism (*n* = 34, 9%), or other (*n* = 1, 0.3%). Participants came from 18 provinces. On average, their demographic distribution resembled that of the 475 initial sample with respect to gender (*X* ^2^_(1)_ = 0.003, *p* = .95); education (*X* ^2^_(5)_ = 0.93, *p* = .97); age (*X* ^2^_(5) (19–29, 30−39, 40−49, 50–59, 60–69, 70 and above)_ = 1.24, *p* = .94); and believer status (*X* ^2^_non-believers vs. believers (1)_ = 0.06, *p* = .81).

### Relations Between Wisdom and Significant Life Learning

The results show that for the analytic sample of 375, participants’ views of the relations between wisdom and significant life learning were distributed in this way among the five types: (a) 191 (51%) participants indicated that their most significant life learning led to their display of wisdom; (b) 91 (24%) participants indicated that their display of wisdom was unrelated to their most significant life learning; (c) 67 (18%) participants indicated that their display of wisdom led to their most significant life learning; (d) 20 (5%) participants indicated that their display of wisdom was part of their most significant life learning; and (f) 6 (2%) participants indicated that their most significant life learning was part of their displays of wisdom.

Thus all five relations exist between displays of wisdom and significant life learning, and the reported frequencies differed from an equal distribution (*X* ^2^_(4)_ = 287.49, *p* < .000). Post hoc tests showed that this distribution had significantly more “learning led to wisdom” (Std. Residual = 13.39, *p* < .000), and significantly fewer “wisdom is part of learning” (Std. Residual = −6.35, *p* < .000) and “learning is part of wisdom” (Std. Residual = −7.97, *p* = .001). The reported frequency of “wisdom led to learning” (Std. Residual = −0.92, *p* = .36) and “no relation” (Std. Residual = 1.85, *p* = .07) did not differ from the equal distribution.

See Table 2 for the participants’ demographic information in the five groups. Results of Chi square tests showed that there were no significant differences in the distribution of participants’ age (*X* ^2^_(24)_ = 26.22, *p* = .34; *X* ^2^_(8) (19–29, 30–39, 40 and above)_ = 14.78, *p* = .06), gender (*X* ^2^_(4)_ = 5.54, *p* < .24), and education level (*X* ^2^_(22)_ = 22.38, *p* = .32; *X* ^2^_(8) (senior high and below, college, master and above)_ = 14.57, *p* = .07) among the five groups.

**Table 2:** Demographic Information on Participants among the Five Categories

Categories	Learning led to wisdom	No relation	Wisdom led to learning	Wisdom is part of learning	Learning is part of wisdom
*N*/*n*	191	91	67	20	6
Female	135	60	38	12	5
Education					
*Elementary and below*	10	2	2	0	0
*Junior high*	7	2	2	3	0
*Senior high*	19	4	8	4	0
*College*	106	64	42	11	5
*Master*	45	18	12	2	1
*Doctoral*	4	1	1	0	0
Age (range)	20–91	19–60	20–92	19–62	20–40
Mean age	34.77	28.24	35.24	36	28
*19–29*	101	66	36	11	4
*30–39*	29	12	11	1	1
*40–49*	18	2	3	1	1
*50–59*	32	10	11	5	0
*60–69*	5	1	3	2	0
*70–79*	5	0	2	0	0
*90–92*	1	0	1	0	0

#### Significant Learning Led to Wisdom

One hundred and ninety-one participants stated that they learned important lessons from their significant experience, and that such learning set the foundation for a later display of wisdom. For example, a 52-year-old male explained that the lessons he learned from his father’s death were to live a healthy lifestyle and treasure his time with family members. His display of wisdom involved embodying an integration of different areas of life by turning down an offer for a high-paid job that would have required him to work in mainland China and live separately from his family. His explanation for the relation between the two experiences was this: “Father’s sudden death let me realize the importance of health and family. Had I not learned from the past experience, I would definitely have chosen that high-paid job” (Participant No. 527, manager, college-level education, non-believer).

#### No Relation

Ninety-one participants indicated that there was no relation between their display of wisdom and their significant life learning, even though most participants’ self-ratings showed that they agreed that what they learned brought them closer to their conceptions of wisdom (*M* = 5.78, SD = 1.16). For example, a 53-year-old man described how his significant life learning came from reconciling with his elder brother from whom he had been estranged after a quarrel many years before. He learned that, no matter how irredeemable the relationship had been, a broken relationship could be mended by initiating short conversations as long as one person was willing to make a move toward the other. His display of wisdom involved embodying an integration of self and other by helping his daughter to see a doctor after spotting a rash on her daughter’s skin that later proved to be shingles, a very painful skin disease. He explained, “My daughter, who did not have a good relationship with me then, thought it was just a common rash and would go away naturally. But I had the experience of having shingles and know that a person will not suffer severe pain in the beginning. I spent much time nagging and eventually persuaded my daughter to see my former doctor who cured my daughter’s disease. My daughter was very grateful to me because she did not suffer any pain” (Participant No. 507, interior decoration worker, junior high level of education, Buddhist). On the third questionnaire, this participant explained, “There were similarities, so in the beginning I thought these two experiences were related, but after thinking more carefully, I did not see how one led to the other or have any relation.”

#### Wisdom Led to Significant Life Learning

Sixty-seven participants indicated that their displays of wisdom led to their significant life learning. Their wisdom was often displayed in their choice of schools, majors, professions, jobs, or partner; such displays of wisdom opened up new doors for their most significant learning in life. For example, a man described that the wisest thing he had done in his life was, after integrating his weaknesses and strengths as well as the positives and negatives of a situation, to choose to attend a specialized school that suited him rather than attending the more prestigious general high school which equipped students to pass the highly competitive national university entrance exam. He explained that although this decision/action went against social convention, his choice freed him from the high academic pressure of the general high school curriculum. As a result, he was motivated to learn more. His significant life learning began when he started to take practica in mechanical engineering. He acquired new ways and habits of learning and eventually earned his doctoral degree in mechanical engineering. Hence, his display of wisdom led to his most significant life learning (Participant No. 440, 43 years old, mechanical engineering, doctoral-level education, folk religion).

A woman explained that as an accountant, she could work in big corporate enterprises, but that her decision to be an accountant in a kindergarten “was a wise decision.” She explained that even though her family had to move to another city, she liked the job and enjoyed her working environment. Her significant learning began after she was settled in her job. She learned from the kindergarten how to treat and teach her children appropriately. She said, “Even though people all say that we should educate our children with love, most my friends and relatives cannot do so without using the rod, and they had difficulty believing that we never used any physical punishment on our children…. Now my children all grown up having stable and warm personality, and they are well-liked by people and among relatives” (Participant No. 652, 52 years old, accounting, college-level education, Christian).

#### Wisdom is Part of Significant Learning

Twenty participants’ significant life learning, such as changing their general attitude and practice toward life, were lengthy processes. Thus, they displayed wisdom while they were still completing their learning. For example, a 44-year-old man described that because his long-expected child turned out to have epilepsy, he had been learning for many years to accept and care for his child. He was still learning as his child grew; nevertheless, he had displayed wisdom through embodying an integration of self and others. Thus far, he had helped many parents whose children also had epilepsy and, in particular, had dissuaded his colleague at work from hiding his epileptic son at home by not sending him to school (Participant No. 529, hairstylist, junior-high-school-level education, Buddhist).

#### Significant Learning is Part of Wisdom

Of the six participants whose responses fell into this category, four were Christian women with college educations. Three displayed their wisdom through believing in God or reaffirming the Christian faith; since such processes usually take a long time, they acquired their most significant life learning within their wisdom process. Hence, their most significant life learning was part of their displays of wisdom.

For example, a 40-year-old woman described that the wisest thing she had ever done in life was, after integrating negatives with positives, deciding to go back to church every Sunday after her husband had been cheated by others and the whole family was seriously in debt. She explained, “10 years ago, my husband helped his younger brother and became his guarantor, but my husband was cheated and was in debt for more than ten million dollars [three hundred thousand U.S. dollars]. Before this event, my husband often arranged family trips on the weekend and it was almost impossible for us to go to church on Sunday. But when this event occurred, we lost focus in life and I was very scared. At that moment I thought of God and felt that He alone can help us…. The moment I was in church, I knew I had made the wisest choice. Within every 1-and half-hour service, I pieced my then-broken self together through God’s love and faced difficulties with positive energy. Life paying back debt was harsh, but we had gone through it and my relationship with my husband was strengthened” (Participant No. 025, teacher, college-level education). Since then, she has kept this spiritual practice. Her most significant life learning was learning to lean on God when she switched to a more highly-paid job so she could help with her family debt, only to find out that her boss cheated her. She learned to refrain from any hasty decisions when in distress but to pray for God’s guidance and let go of any negative emotions. Thus, her most significant life learning was part of her wisdom.

### Main Themes of Significant Life Learning

The results of categorizing participants’ significant learning showed that most participants’ significant life lessons were related to changing their existing general life philosophy or revising attitudes toward life (*n* = 203, 54%). Other main themes of significant life learning included gaining a new insight for life (*n* = 55, 15%), self-related learning (*n* = 21, 5.6%), learning related to friendship and other interpersonal relationship (*n* = 16, 4.3%), learning related to romantic relationship (*n* = 13, 3.4%), and family-related learning (n = 9, 2.4%).

#### Possible Similarities and Differences Between Different Types of Learning

Although not the main focus of this study, it is worth exploring whether these data indicate that significant life learning has different characteristics when it is related to wisdom and when it is not, and whether learning that was preceded by wisdom differs from learning that led to wisdom. I begin this inquiry with some basic statistics.

##### Between Wisdom-related Learning and Learning Unrelated to Wisdom

Participants’ self-ratings for wisdom did not differ significantly on average between wisdom-related learning (*M*_*wisdom*–*related*_ = 5.80, SD = 0.96) and non-wisdom-related learning (*M*_*non*–*wisdom*–*related*_ = 5.77, SD = 0.98; *t*_(373)_ = 0.18, *p* = .86). Nor did the learning unrelated to wisdom differ from wisdom-related life learning in the distribution of the main learning categories (*X* ^2^_(5)_ = 8.87, *p* = .11). However, analysts/raters’ wisdom ratings were significantly higher for wisdom-related learning (*M*_*wisdom related*_ = 6.44, SD = 0.80) than learning that participants perceived as unrelated to wisdom (*M* = 6.28, SD = 0.92; *t*_(353.32)_ = 2.41, *p* < .05, *d* = 0.32, 95% CI [0.03, 0.29]).

##### Between Learning that Led to Wisdom and Learning that Was Preceded by Wisdom

Participants’ self-ratings for wisdom in learning that was preceded by wisdom (*M*_*learning led by wisdom*_ = 5.93, SD = 0.91) were a bit higher on average than learning that led to wisdom (*M*_*learning led to wisdom*_ = 5.76, SD = 0.99), but the difference did not reach a significant level (*t*_(256)_ = −1.17, *p* = .24). Analysts/raters’ wisdom ratings for these two kinds of life learning did not differ significantly (*M*_*learning led to wisdom*_ = 6.44, SD = 0.81; *M*_*learning led by wisdom*_ = 6.42, SD = 0.78; *t*_(647)_ = 2.39, *p* = .81). Nor did the two kinds of wisdom-related learning differ in the distribution of the main learning categories (*X* ^2^_(5)_ = 6.71, *p* = .24).

## Discussion

The results of this study show that, as hypothesized, all five relations exist in people’s recollection of their most significant life learning and their display of wisdom. As life is an ongoing process, it is quite possible that the perceived relation of the two experiences may evolve and change as life goes on. Nevertheless, the findings demonstrate that the relations between wisdom and significant life learning are complex and intertwined.

### Learning that Fosters Wisdom

The significantly high frequency (51% of the analytic sample) of the “learning led to wisdom” group in this study aligns with what has been proposed (Ardelt 2010; Yang 2014b) that life learning fosters wisdom. It also shows that learning from significant life experience is a more commonly observed mechanism of developing wisdom. The findings touch on one possible way to foster wisdom: examining what we learn from our significant life experience, implementing the learned lessons, and evaluating the effects of our implementation.

### Wisdom-fostered Learning

The fact that a sizable number of respondents (*n* = 67) felt that wisdom can open doors to a whole repertoire of significant life learning suggests the positive function that wisdom may have in people’s lives. Moreover, most of the descriptions of such “wisdom-fostered learning” show that applying the lessons learned often generated positive effects at least for the self, but often for others as well. Through the positive effects generated by the display of wisdom and by the positive effects generated by the application of wisdom-fostered learning, a person’s development is set onto an even more positive direction. It is perhaps by this “double-dosed” positive effects that wisdom-fostered learning is set apart from the kind of life learning acquired from unwise decisions and actions.

In addition, the large number of young people in this category suggested that the positive functions can be observed in the young as well as in the old. The findings also suggest that wisdom-fostered learning can occur in both informal and formal educational contexts. The substantial number of the “wisdom led to learning” group implies that we may also foster wisdom by looking deep into our own selves and noting what we did that was wise (Bassett 2006), since we often gain insight and strength to move forward by learning from what we have done right in the past.

### Other Findings

Even though descriptions of wisdom as part of learning and learning as part of wisdom were few, the findings show that some people perceive that wisdom may entail learning and vice versa. Interestingly, most descriptions concerning learning as part of wisdom came from Christian women. However, the sample size was too small to draw any meaningful conclusion. Further studies could explore the role that religion or gender may play in the perception of the relations.

In addition, almost one-quarter of participants indicated that there was no relation between the two experiences, even though most of them also indicated that what they learned from significant experiences brought them closer to their conceptions of wisdom. Perhaps this is so because they have not implemented what they learned to real life or because their implementation has not generated positive effects for themselves and others. Future studies are needed to explore further the reasons behind the perception that wisdom and significant life learning are unrelated.

Finally, results of preliminary analysis of learning showed that most significant life learning was related to revising one’s life philosophy, and that analysts/raters tended to give higher wisdom ratings to life lessons that participants indicated related to wisdom than those that were not. As suggested earlier, future efforts should employ more systematic ways to examine whether there is any fundamental difference between learning led to wisdom and learning led by wisdom, as well as what kinds of situations cause life learning and displays of wisdom.

### Limitations

Some limitations of this study can be noted. First, although the sample of this study was diverse in terms of age, educational level, gender, and religion, it was not representative of the population in Taiwan. Second, this study administered the questionnaires in a constant order, with the life-learning questionnaire always coming first, and hence may have generated a certain priming effect on the participants’ search for a wisdom event. Future research should counterbalance the order of the first two questionnaires in conducting similar studies. Third, while conducting the study mostly through email may allow the researchers to reach a wider population, it also allowed participants to go back and read their first two questionnaires as they were working on the third one. Thus, future research should use an online survey that puts all three questionnaires together but does not allow participants to go back and read their response to the previous questionnaires. Finally, this study presupposed that learning and wisdom are two different processes, and hence did not consider the possibility that the display of wisdom is the most significant life learning. Future research should take this extra relation into consideration.

## Conclusions and Suggestions

This preliminary effort has showed that ordinary people can and do display wisdom, as defined by the process theory of wisdom. In addition, the results suggesting that our own displays of wisdom can be both the motivator and outcome of learning may have important implications for adult learning and education. This would be particularly true concerning informal learning, including everyday learning, biographical learning, as well as learning from one’s life history (Jarvis 2009).

More significantly, the findings shed light on how wisdom and the learning acquired from significant life experiences might foster individual development. The spiral effects co-created by applying significant life lessons and displays of wisdom can affect individuals’ life-span development in positive ways. This could have important implications for counseling, positive psychology, life-span development, and aging.
